# Nucleocytoplasmic shuttling of SOX14A and SOX14B transcription factors

**DOI:** 10.18632/oncotarget.15134

**Published:** 2017-02-07

**Authors:** Zhen-Yu She, Wan-Xi Yang

**Affiliations:** ^1^ The Sperm Laboratory, College of Life Sciences, Zhejiang University, Hangzhou 310058, China

**Keywords:** SOX14, HMG box, nuclear localization signal, nuclear transport, nuclear export

## Abstract

The nucleocytoplasmic shuttling of SOX transcription factors play a crucial role in the regulation of SOX protein functions during development. In this study, we have demonstrated two nuclear localization signals in the HMG box of *Eriocheir sinensis* SOX14A and SOX14B. These two conserved nuclear localization signals mediate nuclear transport. The N-termini nuclear localization signal mediates the calmodulin-dependent pathway and the C-termini nuclear localization signal interacts with the importin-β pathway. The targeted deletion of nuclear localization signals of SOX14A/B dramatically inhibits the nuclear accumulation. We have first time revealed a non-classic nuclear export signal in the HMG box of *E. sinensis* SOX14A/B proteins is responds to leptomycin B. *E. sinensis* SOX14A/B is transported from the nucleus to the cytoplasm via a CRM1-dependent nuclear export pathway. And *E. sinensis* SOX14A/B are not belong to the subgroup E SOX proteins. Furthermore, these findings could shed a light on the mechanisms involved in the nuclear export of SOX proteins. The imperfect nuclear export signal on other SOX proteins, rather than just those of the SOXE group, may also be functional for nuclear export.

## INTRODUCTION

The SOX (SRY-related HMG box) family transcription factors have been discovered throughout the animal kingdom. They occur in diverse taxa, from flies to nematode worms, to insects, to mammals, and are well-known regulators of a variety of developmental processes, particularly relating to neural development, sex determination and testis development, chondrogenesis and cell fate decision [1–3]. Research on SOX family proteins began with the identification of *SRY* (the sex determining region of chromosome Y), which is the founding member of the SOX family of transcription factors. It functions as a master regulator of mammalian sex determination pathways and mediates the differentiation of Sertoli cell lineages in the bipotential genital ridge in both humans and mice [4–8]. In vertebrates, the SOX family proteins are comprised of more than 20 *Sox* genes members which can be further subdivided into groups A-J based on the sequence alignments of the evolutionarily conserved HMG box domain [9–10]. Approximately 10 *Sox* genes have also been identified and documented as present in many invertebrate species including *Drosophila melanogaster*, *Apis mellifera*, and *Caenorhabditis elegans* and other lower metazoans. However, the developmental functions of invertebrate *Sox* genes remain largely unknown [11–12].

SOX proteins are characterized by an evolutionarily conserved high mobility group (HMG) DNA-binding domain which can specifically recognize and bind to the consensus sequence (A/T)ACAA(T/A) or to related sequence motifs in the minor groove of the DNA. Such a binding process induces a topological 60-90° bend of the DNA helix in order to transactivate the transcription of target genes [7, 13–15]. Accumulating evidence relating to many studies indicates the emerging elucidation of multiple roles of the 79-amino-acid HMG box. This includes its role in the DNA binding and bending of biochemical properties as well as in protein-protein interactions with partner proteins in diverse cellular events [13, 16, 17].

The nucleocytoplasmic transport of protein molecules (>40 kDa) across the nuclear envelope through the nuclear pore complexes is a highly dynamic activity occurring between the nucleus and the cytoplasm. It is required for the complex regulation of diverse cellular functions [18–20]. Both human SRY and SOX9 contain a bipartite nuclear localization signal (NLS) motif and a basic cluster NLS motif, located in the N-terminal and C-terminal of the HMG box, respectively [21–23]. The N-terminal NLS resembles two highly basic consensus sequences separated by 9-12 residues. It interacts with calcium-activated calmodulin to increase the nuclear import of SRY and SOX9 and subsequent transcriptional activities [24]. The C-terminal NLS of SRY interacts with importin-β and mediates nuclear localization through a Ran-GTP dependent pathway [25]. The SOX subgroup E proteins, including SOX8, SOX9 and SOX10, harbor a well-characterized leucine-rich motif. This is a nuclear export signal (NES) located in the HMG box [20]. The nuclear export signals in SOX9 and SOX10 are recognized by CRM1 and can be specifically inhibited by leptomycin B (LMB) [26–27]. The NLS/NES, within the HMG box, are essential for the subcellular localizations and nuclear import/export equilibrium of SOXE proteins. Loss-of-function studies have suggested that the HMG box stimulates the nucleocytoplasmic shuttling properties of SOX proteins and is responsible for the highly dynamic regulation network of SOX proteins during development [7, 20, 28].

Recently, we studied the roles of SOX proteins in Chinese mitten carb *E. sinensis*. Surprisingly, we found that *E. sinensis* SOX14A/B proteins shuttle between the nucleus and cytoplasm, and can response to LMB. These results suggested that SOX14A/B may have a novel nuclear export signal. To date, however, there is no crustacean cell lines available for the study of *E. sinensis* Sox proteins. Due to the high transfection efficiency and easy genetic methods of the human embryonic kidney HEK293T cell line, we choose it as our model cell line in this study.

In this study, we found that both *Eriocheir sinensis* SOX14A and SOX14B contain two nuclear localization signals and a nuclear export signal, localized in the HMG box, which mediate nucleocytoplasmic shuttling of these two transcriptional factors. In addition, we demonstrated for the first time that a non-classic nuclear export signal that occurs in the HMG box of SOX14A/B proteins but which lies outside of the subgroup E SOX proteins, is functional and that the nuclear export of SOX14A and SOX14B can be blocked by LMB. This have indicated that the nuclear export of SOX14A and SOX14B is regulated by a CRM1 dependent mechanism. Here we reveal the molecular mechanisms involved in the nucleocytoplasmic shuttling of SOX14A and SOX14B in cultured cells and provide the first evidence that the hydrophobic NES, located on these two SOX proteins are functional.

## RESULTS

### Subcellular distribution of *Eriocheir sinensis* SOX14A and SOX14B in cultured cells

**Figure 1:**
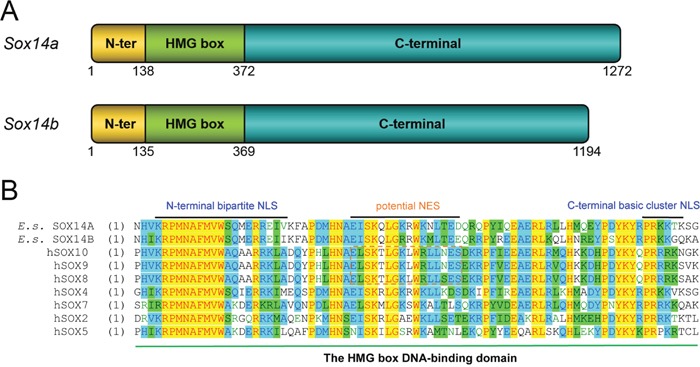
The gene structures of *E. sinensis Sox14a* and *Sox14b* and the multiple sequence alignment of the HMG box of SOX14A/B and other representative SOX proteins **A**. Schematic representations of the *E. sinensis Sox14a* and *Sox14b* genes. Both *E. sinensis* SOX14A and SOX14B are composed of three major domains: the N-terminal domain (indicated in yellow), the HMG DNA-binding domain (indicated in green), and the C-terminal transcriptional domain (indicated in blue). The numbers below the models indicate the positions of each functional domain in *E. sinensis Sox14a* and *Sox14b* genes, respectively. **B**. Multiple sequence alignments of the HMG box of *E. sinensis* SOX14A and SOX14B with other typical SOX proteins in human representatives for each SOX group. About 81 amino acids of the HMG box are aligned and shown in the figure. The N-terminal bipartite NLS and the C-terminal basic cluster NLS are indicated in the N-termini and C-termini of the HMG box. In the middle of the HMG box, the conserved leucine-rich nuclear export signal of the SOXE groups, including SOX8, SOX9 and SOX10, are denoted with the dashed box. *Homo sapiens* SOX2 is the classical member of SOXB group. *Homo sapiens* SOX4 represents SOXC group. *Homo sapiens* SOX5 represents SOXD group. *Homo sapiens* SOX7 represents SOXF group. In this figure, yellow indicate most conserved region, green indicate conserved region, blue indicate less conserved region. The corresponding names and NCBI Accession Numbers in GenBank are listed as follows: *Eriocheir sinensis* SOX14A (KC896287), *Eriocheir sinensis* SOX14B (KC896286), *Homo sapiens* SOX8 (AAH31797), *Homo sapiens* SOX9 (CAA86598), *Homo sapiens* SOX10 (CAG38808), *Homo sapiens* SOX2 (NP_003097), *Homo sapiens* SOX4 (AAH72668), *Homo sapiens* SOX5 (CAG32994), *Homo sapiens* SOX7 (CAC84226).

Our clone of the *Eriocheir sinensis Sox14a* (GenBank accession no. KC896287) and *Sox14b* genes (GenBank accession no. KC896286) together with the structure and functional domains of these two transcription factors are shown in Figure 1A (Supplementary Figure 1A, 1B). The amino acids of the HMG box in *Eriocheir sinensis* SOX14A and SOX14B and *Homo sapiens* SOX14 are highly conserved. Multiple sequence alignments have indicated that both the HMG box of SOX14A and SOX14B are evolutionarily conserved with other SOX proteins in both *H. sapiens* and *M. musculus* (Figure 1B, Supplementary Figure 2A, 2B). Compared with other reported NLS sequences (Supplementary Figure 2B), we found that both *Eriocheir sinensis* SOX14A and SOX14B proteins contain two putative nuclear localization signals at either side of the HMG box [21, 29]. The nuclear localization signals are comprised of an N-termini bi-partite NLS, which contains two groups of basic residues separated by 9-11 residues, and a monopartite C-termini NLS, which is characterized by a short stretch of 4-5 basic residues (Figure 1B). In addition, a candidate nuclear export signal is also located in the HMG box of SOX14A and SOX14B. Alignments of the NES sequences with other representative leucine-rich NESs within the HMG box of SOX9 and SOX10 have suggested that the NES signal in SOX14A and SOX14B are potential but not valid (Figure 1B) [26, 27].

**Figure 2:**
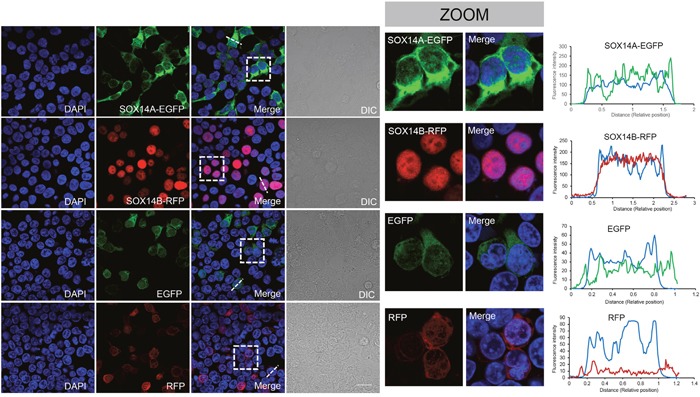
The subcellular distributions of *E. sinensis* SOX14A and SOX14B in 293T cells The 293T cells were transiently transfected with SOX14-EGFP, SOX14B-RFP, EGFP, RFP expression vectors and maintained at 37°C for 24h. The cells were fixed and the nuclei were stained by DAPI (blue). Differential interference contrast (DIC) microscopy was used to indicate the shapes of the cells. The scale bar represents 10 μm. Subcellular location of SOX14A-EGFP in transiently transfected 293T cells. SOX14A-EGFP proteins were expressed in both the nucleus and cytoplasm. Subcellular distribution of SOX14B-RFP in 293T cells. SOX14B-RFP proteins mainly accumulate in the nucleus in most cells, where very few remain in the cytoplasm. In control, the transient expression of EGFP alone shown the cytoplasmic and nucleoplasmic signals without special locations. In control, RFP also showed a dispersed distribution in 293T cells. The fluorescence intensities of SOX14A/B-fluorescent protein transfected cells were analyzed by the ImageJ software. The X axis means the relative position of the cell. The Y axis means the relative fluorescence intensities. The representative cells and analyzed position were indicated by the white dot line.

To investigate the subcellular distributions of SOX14A and SOX14B, we used the HEK293T cell as a model to study the intracellular locations of SOX14A and SOX14B. We genetically fused the full-length transcripts of SOX14 and SOX14B in frame with the open reading frame of the enhanced green fluorescent protein (EGFP) and red fluorescent protein (RFP), respectively, using the pCMV-N-Flag vector (Supplementary Figure 3A, 3C). After transient transfection of the SOX14A-EGFP and SOX14B-RFP fusion transcripts in 293T cells for 24 h, we collected and analyzed the subcellular locations of SOX14A and SOX14B by direct immunofluorescence assays using a confocal laser scanning microscope (Supplementary Figure 4). The fluorescence intensities of EGFP/RFP/DAPI were analyzed in representative cells to indicate the expression pattern of transfected proteins. Strikingly, we found that the subcellular distributions of SOX14A and SOX14B proteins are different in cultured cells. In the cultured 293T cells, SOX14A proteins were distributed in both the cytoplasm and nucleus, and in a relative predominantly cytoplasmic pattern (Figure 2, Supplementary Figure 4C). In contrast, SOX14B exhibited an almost exclusively nuclear distribution pattern, with little distribution within the cytoplasm (Figure 2, Supplementary Figure 4C). In the control experiments, the transfections of EGFP or RFP alone resulted in both cytoplasmic and nucleocytoplasmic signals. The fluorescent proteins did not affect the distribution of fusion proteins (Figure 2).

### The HMG box are required for the nuclear import of *E. sinensis* SOX14A and SOX14B

**Figure 3:**
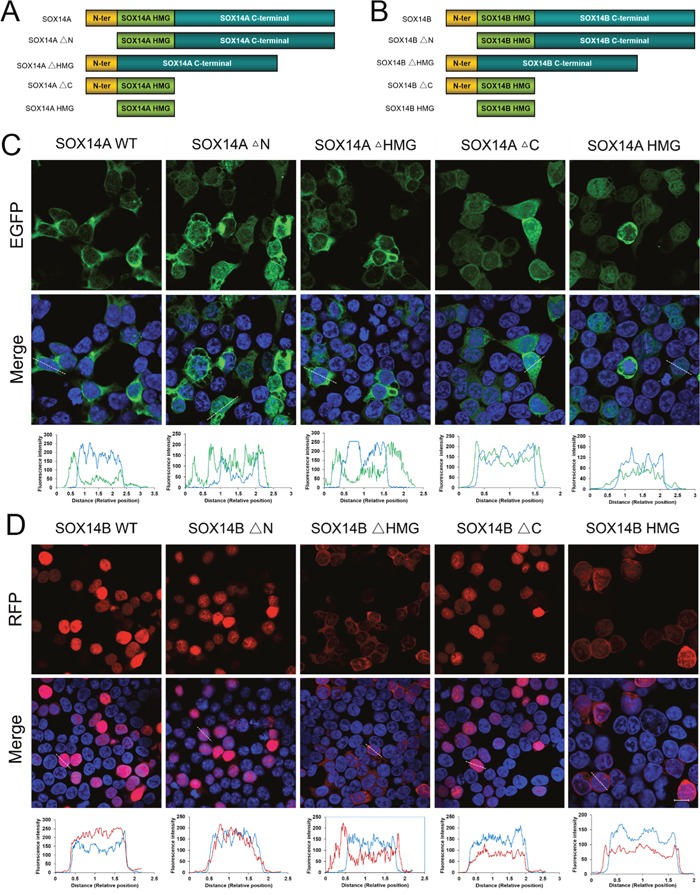
The HMG box are involved in the nuclear import of *E. sinensis* SOX14A and SOX14B proteins **A/B**. The *E. sinensis* SOX14A/B full-length (wild type, WT) or mutant fusion constructs used in this study. The deletion of N-terminal, the HMG box and the C-terminal are indicated as SOX14A/B ΔN, ΔHMG and ΔC, respectively. **C**. 293T cells were transiently transfected with the SOX14A wild type or mutant EGFP-fusion plasmids and then cultured for 24h and then analyzed by the fluorescence microscopy. **D**. Similarly, the plasmids encoding the SOX14B wild type or mutant fusion proteins were transfected into 293T cells for 24h. The fluorescence intensity of representative cells were analyzed and indicated in the table. The blue line indicates the fluorescence intensity of DAPI, the green line indicates the fluorescence intensity of EGFP-fusion proteins, the red line indicates the fluorescence intensity of RFP-fusion proteins. Confocal images were then taken under the same exposure intensity by Zeiss CLSM 710 microscopy (Carl Zeiss). The SOX14A-EGFP (green), SOX14B-RFP (red), and DAPI (nucleus, blue) were shown in the Figure 3C, 3D. DIC microscopy was applied to visualize the cell morphology. The scale bar represents 10 μm.

To investigate the molecular mechanisms involved in the subcellular distributions of SOX14A and SOX14B proteins in more detail, we deleted either the N-terminal domain (ΔN), or the HMG box (ΔHMG), or the C-terminal domain (ΔC) of both SOX14A and SOX14B proteins (Figure 3A, 3B, Supplementary Figure 4B). Compared with the wild type containing a full-length SOX14A, the deletion of the N-terminal domain or the C-terminal domain of SOX14A did not affect the distribution of SOX14A in the nucleus and cytoplasm (Figure 3C). Similarly, the nuclear localizations of the SOX14B ΔN mutant or ΔC mutant fusion proteins remained intact in the transfected 293T cells (Figure 3D). In contrast, the deletion of the HMG domain of SOX14A and SOX14B significantly disrupted the nuclear localizations of SOX14A and SOX14B fusion proteins. Here, fluorescent signals were seen as excluded from the nucleus, as confirmed by differential interference contrast microscopy (Figure 3C). Whilst consistent with the results in control experiments on wild type SOX14A/B, the HMG domains of SOX14A and SOX14B have shown a similar subcellular localization with those of full-length SOX14A and SOX14B (Figure 3C, 3D).

### The nuclear localization signals (NLSs) in the HMG box of *E. sinensis* SOX14A/B are crucial for nuclear import

**Figure 4:**
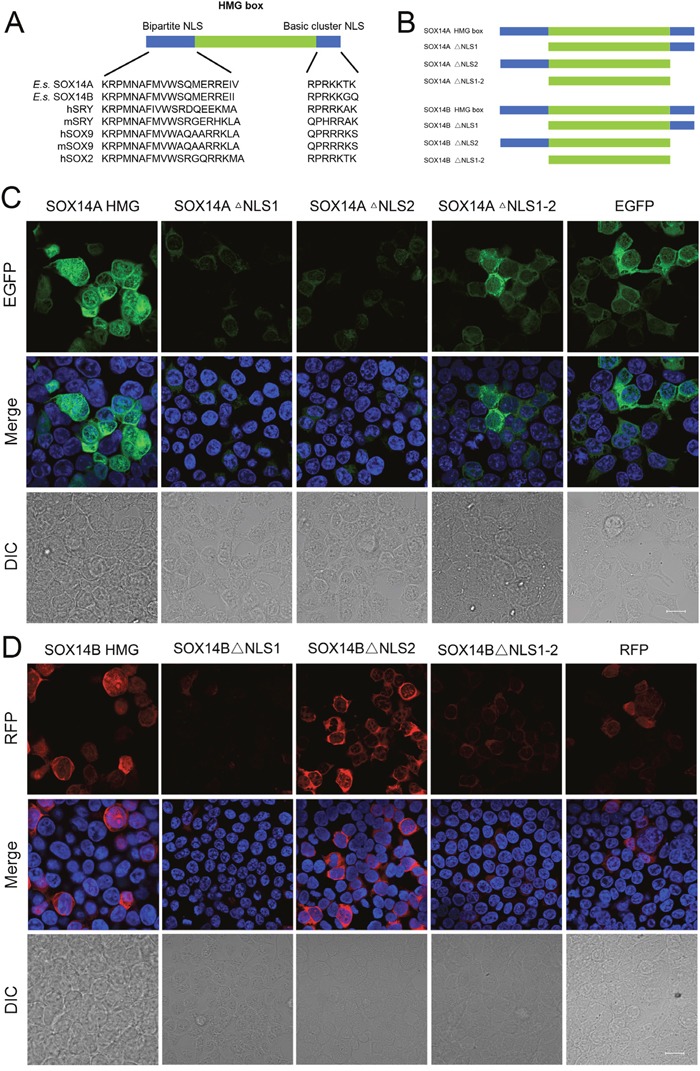
The nuclear localization signals in the HMG box of *E. sinensis* SOX14A/B are responsible for nuclear import **A**. Alignment of conserved nuclear localization signals in the N-termini and C-termini of the HMG box. Comparison of the N-termini and C-termini NLS of SOX14A and SOX14B with the corresponding sequences of other SOX proteins, including hSRY, mSRY, hSOX9, mSOX9, hSOX2. h indicates *Homo sapiens*. m indicates *Mus musculus*. **B**. The SOX14A/B HMG box or mutant fusion constructs used in this study. The deletion of N-termini NLS, C-termini NLS and both these two NLSs are indicated as SOX14A/B ΔNLS1, ΔNLS2 and ΔNLS1-2, respectively. **C and D**. 293T cells were transfected with the SOX14A/B HMG box, ΔNLS1, ΔNLS2 or ΔNLS1-2 expression plasmids, respectively. After 24h expression, cells were fixed and stained with DAPI for fluorescence microscopy. The EGFP (green), RFP (red) and DAPI (nucleus, blue) were shown in the confocal images. The scale bar represents 10μm. The fluorescence intensity of representative cells were plotted by ImageJ software and the quantitative analyses were indicated in the table. The blue line indicates the fluorescence intensity of DAPI, the green line indicates the fluorescence intensity of EGFP-fusion proteins, the red line indicates the fluorescence intensity of RFP-fusion proteins.

We then focused upon the precise roles of the HMG box in the intracellular distributions of SOX14A and SOX14B. We found that two highly conserved NLSs located in the HMG domain, one at the N-termini and another at the C-termini (Figure 4A). To demonstrate the functions of these two NLSs, the subcellular localizations of different SOX14A and SOX14B mutant protein constructs were examined in the 293T cells (Figure 4B, Supplementary Figure 4B). Strikingly, the nuclear translocations of the HMG box of *E. sinensis* SOX14A and SOX14B were largely inhibited when the N-termini NLS of the HMG box were deleted, as shown by SOX14A/B ΔNLS1 fusion protein (Figure 4C, 4D). A similar, but less pronounced and more partial loss of the nuclear localization of fusion proteins resulted from the deletion of C-termini NLS of the HMG box (SOX14A/B ΔNLS2) (Figure 4C, 4D). As shown in Figure 4C, 4D the deletion of both the N-termini NLS and the C-termini NLS of the HMG box of *E. sinensis* SOX14A and SOX14B led to a complete loss of the nuclear translocation of mutant fusion proteins.

### The nuclear export of SOX14A and SOX14B depend on CRM1 and can be inhibited by LMB

**Figure 5:**
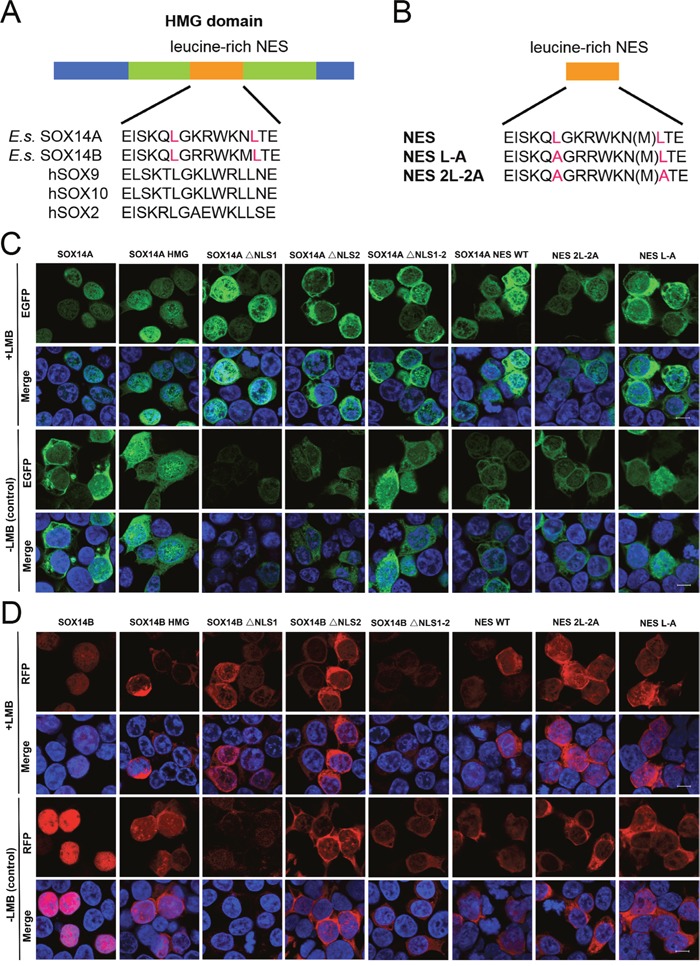
The nuclear export of *E. sinensis* SOX14A and SOX14B are dependent on the NES signal and can be inhibited by LMB **A**. Alignment of the potential NES in SOX14A and SOX14B with the conserved leucine-rich NES sequences of hSOX9, hSOX10 and hSOX2. **B**. Schematic representations of the different mutant NES sequences in the expression plasmid constructs, which are indicated as SOX14A/B NES, NES L-A and NES 2L-2A, respectively. **C and D**. 293T cells were transiently transfected with SOX14A/B WT, HMG box, ΔNLS1, ΔNLS2, ΔNLS1-2, NES, NES L-A and NES 2L-2A EGFP (green) fusion plasmids and cultured for 24h. Before harvest, the leptomycin B (5.0 ng/ml; +LMB) were added into the cells for 3h before fixing. In the control, no leptomycin B (-LMB) were used. 293T cells were stained with DAPI (blue) to visualize the nucleus, and the EGFP/RFP subcellular distributions were analyzed by confocal laser scanning microscopy for subsequent fluorescence analyses. The scale bar represents 10μm.

Finally, we identified a potential nuclear export signal in both of the HMG domains of SOX14A and SOX14B (Figure 5A). The classic NES sequences identified in SOX9 and SOX10, as revealed in previous studies exhibit the common feature Φ_1_-X_2-3_-Φ_2_-X_2-3_-Φ_3_-X-Φ_4_ (where Φ=L, I, V, F or M and X represents any amino acid). However, in other subgroups outside the subgroup E, the residue Φ_3_ is absent [20, 28, 34]. There is a long-standing controversy regarding whether the NES sequences in other SOX proteins are functional and sufficient to facilitate nuclear export. It is noted that even these putative NES sequences can bind to the CRM1/exportin1 with a low affinity [28, 30]. Further studies are required to confirm any such roles of the putative non-conventional NES sequences in other SOX proteins. Our results from multiple sequence comparisons do, however, provide strong evidence that *E. sinensis* SOX14A and SOX14B do harbor a potential NES sequence (Φ_1_-X_2-3_-Φ_2_-X_5-6_-Φ_4_), which is a common consensus of non-conventional NES sequences where Φ3 is absent (Figure 5A).

**Figure 6:**
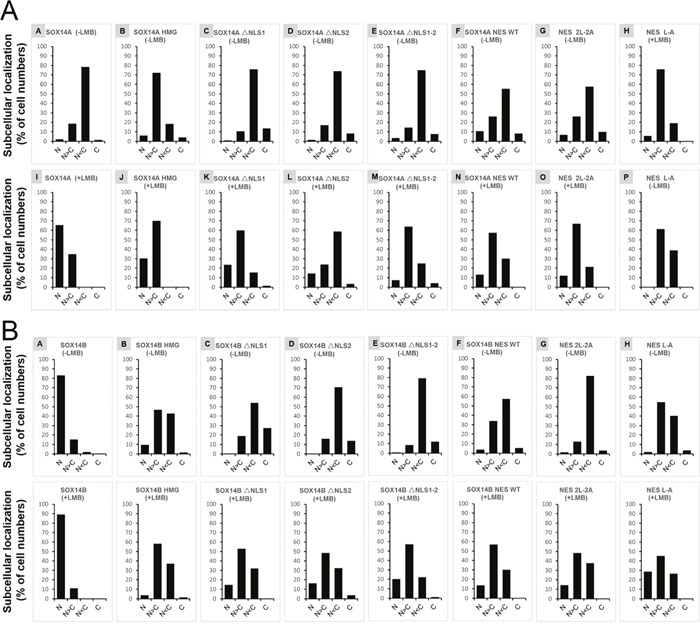
The ratios of different subcellular locations of SOX14A/B-EGFP/RFP fusion proteins in 293T cells **A and B**. Quantification of the total cell numbers of wild type or mutant SOX14A/B-EGFP/RFP locations in the nucleus and the cytoplasm from three independent experiments. The total numbers of the different types were shown as the percentage (%) in the tables. Four different kinds of cells are counted and the ratio of each kind are indicated, including nucleus (N), nucleus more than cytoplasm (N>C), cytoplasm more than nucleus (C>N), cytoplasm (C). Total cells from three independent experiments were collected and analysis for the distribution of fluorescent proteins in nucleus or cytoplasm by ImageJ software and Microsoft Excel tools.

To further address this question, we generated different SOX14A/B HMG box mutant fusion protein constructs and examined the subcellular locations of each fusion protein in 293T cells (Figure 5B). The full-length SOX14A-EGFP fusion proteins were located in both the nucleus and the cytoplasm (Figure 5C), but when the nuclear export was inhibited by LMB, SOX14A-EGFP proteins were accumulated in the nucleus (Figure 5C). This result indicates that SOX14A proteins are able to transport from the nucleus to the cytoplasm via the CRM1-mediate mechanism. The SOX14A HMG box fusion proteins also show a similar phenotype. SOX14A ΔNLS1, ΔNLS2, and ΔNLS1-2 are all seen to be accumulated in the nucleus, but upon deletion of nuclear localization signals the nuclear import of the fusion proteins is partially decreased (Figure 5C, Figure 6A). These results consolidate the existence of potential CRM1-dependent nuclear export signals in the HMG box of SOX14A.

The full-length SOX14B-RFP proteins were mainly located in the nucleus of transfected 293T cells (Figure 5D). The SOX14B-RFP were restricted in the nucleus when treated with LMB (5.0 ng/ml) for 3 h before fixing (Figure 5D). We found that the deletion of the nuclear localization signals could dramatically decrease the nuclear import of SOX14B ΔNLS1, ΔNLS2, and ΔNLS1-2 fusion proteins (Figure 5D). When these cells were treated with LMB, which inhibits the CRM1-mediated nuclear export pathway, these fusion proteins displayed a tendency to accumulate in the nucleus of cultured cells (Figure 5D, Figure 6).

### The nuclear export signal (NES) located in the HMG box of SOX14A/B is responsible for nuclear export

To narrow down the nuclear export activity to the putative NES site in the HMG box of SOX14A (EISKQLGKRWKNLTE), we constructed both the fusion proteins that fused the synthetic double-stranded oligos corresponding to this NES site and the two mutant NESs, comprised of either substitutions of alanine for the two leucines or for the single leucine (denoted as NES WT, NES 2L-2A, and NES L-A, respectively) (Figure 5B, Supplementary Figure 1B). The SOX14A NES wild-type EGFP fusion proteins were expressed in both the nucleus and cytoplasm, and were also mainly localized in the nucleus beyond treatment with LMB. This suggests that SOX14A NES can respond to the LMB and is required for nuclear export. When all leucines in NES were substituted for the alanines (SOX14A NES 2L-2A), the amounts of nuclear export were decreased. A similar result can also be seen in the single substitution NES L-A (Figure 5C). This indicates that the leucine stimulates the nuclear export of SOX14A.

We also targeted the nuclear export activity to the potential NES sequence in the HMG box of SOX14B (EISKQLGKRWKMLTE), and we constructed a series of SOX14B NES WT, NES 2L-2A, and NES L-A mutant fusion proteins to test the function of the NES sequence in SOX14B and the roles of leucines in the NES (Figure 5B). Similarly, SOX14B NES-RFP fusion proteins were widely expressed in the nucleus and cytoplasm. They then gradually accumulated in the nucleus upon the addition of LMB (5.0 ng/ml) for 3h. When both of the leucines were replaced by the alanines in SOX14B NES, the amount of SOX14B NES 2L-2A RFP fusion proteins were slightly decreased (Figure 5D, Figure 6B). Similar results were also shown in single substitution SOX14B NES L-A (Figure 5D). Both these two leucines in NES are therefore necessary for the nuclear export of SOX14B.

## DISCUSSION

### The dynamic shuttling of SOX proteins in development

The highly dynamic and complex shuttling of SOX transcription factors between the nucleus and the cytoplasm is required for the functions of SOX proteins in development [28, 33–39]. Previous studies have shown that the nuclear import of SOX9 proteins is mediated by two independent pathways: the N-termini NLS is involved in a calmodulin-dependent nuclear import pathway and the C-termini NLS mediates the importin-β nuclear import pathway [21, 24, 37]. Further research into the nuclear import mechanisms of SOX proteins revealed that nearly all of SOX protein members contain these two conserved nuclear localization signals at the either end of the HMG box and these two NLSs contribute to the subcellular location and nuclear transport of SOX proteins during many developmental events [20, 25, 29].

SOX proteins recognized and binding by the importins are complex and regulated the nuclear localization signals locating at the both ends of the HMG box. Previous study suggest that there are several parallel import pathways for the nuclear import of SOX2 proteins [30]. Exportin 4 functions as efficient nuclear import receptor of SOX2 and SRY proteins. Meanwhile, Imp9 and the Imp-β/7 heterodimer also stimulate the nuclear import of SOX2 [30]. During neural differentiation, Imp-β/α3 and Imp-β/α5 are involved in the nuclear import of SOX2 proteins in differentiated neural cells. In undifferentiated embryonic stem cells, Imp-β family proteins could mediate the nuclear import of SOX2 [31].

### The HMG box and two NLSs stimulate the nuclear import of *E. sinensis* SOX14A/B

In this study, we have revealed the subcellular distribution of *E. sinensis* SOX14A and SOX14B in the nucleus and the cytoplasm in cultured 293T cells. We have also demonstrated that the HMG box on SOX14A and SOX14B are crucial to the nuclear import of these two transcription factors. These different locations of EGFP and RFP may be due to the different nuclear import efficiencies of the HMG box in SOX14A and SOX14B. Target deletions of the N-termini NLS or the C-termini NLS largely excluded the SOX14A and SOX14B proteins from the nucleus. Combined depletion of the N-termini NLS and C-termini NLS significantly decreased the nuclear accumulation of SOX14A and SOX14B in the cultured cells. These data indicate that both the N-termini bi-partite NLS and the mono-partite C-termini NLS are involved in the nuclear import of SOX14A and SOX14B, but each to a different extent.

**Figure 7:**
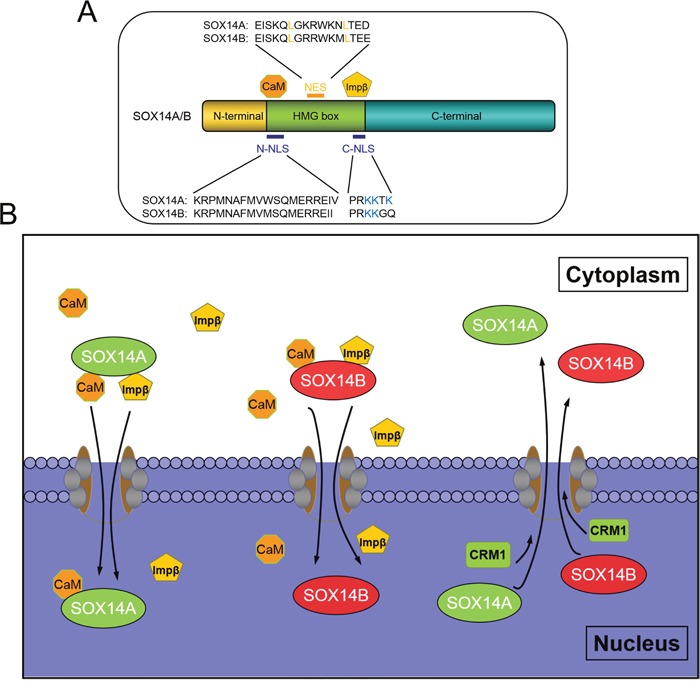
The molecular mechanisms involved in the nucleocytoplasmic shuttling of *E. sinensis* SOX14A and SOX14B **A**. The HMG box is necessary for nuclear import of SOX14A and SOX14B. The N-termini NLS mediate the nuclear translocation of SOX14A/B through a calmodulin-dependent pathway and C-termini NLS interacts with importin-β pathway to stimulate nuclear transport. Both *E. sinensis* SOX14A and SOX14B contains an imperfect hydrophobic NES, which is sensitive to the CRM1-specific inhibitor LMB. **B**. The classical pathways of the nucleocytoplasm transport of SOX9/10 are well established. The calmodulin and importin-β can facilitate the nuclear import of SOXE group protein SOX9 and SOX10. And the classical leucine-rich sequence of HMG box is necessary and sufficient to export the SOX9/10 protein out of the nucleus. In our study, we found that the N-termini NLS and the C-termini NLS of SOX14A/B mediates the nuclear import across the nuclear pore complex, this could be mediated by calmodulin and Importin-β. Strikingly, we found a new nonconventional nuclear export pathway which activates SOX14A and SOX14B transport from the nucleus to the cytoplasm through the CRM1-dependent pathway. We demonstrated that the leucine-rich NES sequence located in the HMG box is response to leptomycin B. This indicated that the NES signals that required for nuclear export are not only existed in the well-known SOX9/10. The other SOX proteins, such as SOX14A and SOX14B, also have the functional NES signals.

Our result indicates both the evolutionarily conserved NLSs, either side of the HMG box, are required for nuclear translocation of SOX14A and SOX14B (Figure 7A, 7B). In summary, these data suggests that the N-terminal domain and the C-terminal domain of SOX14A and SOX14B are not involved in the nuclear localization of SOX14A and SOX14B and that the HMG domains are required for the nuclear import of these two proteins.

### The leucine-rich NES in SOX14A/B activates the CRM1-dependent pathway to increase nuclear export

CRM1 belongs to the importin-β superfamily of nuclear transport receptors and recognizes a large number of nuclear transcription factors, ribosomal subunits or mRNA substrates [30, 34]. LMB, a *Streptomyces* metabolite, can directly bind to CRM1 and disrupt the interactions between CRM1 and leucine-rich or hydrophobic NES in order to inhibit the CRM1 mediation of nuclear export [35–39]. To date, the best characterized molecular mechanism involved in the nuclear export of the SOX proteins is the CRM1-dependent pathway. The CRM1 nuclear export receptor can recognize and bind to the leucine-rich NES found in the subgroup E proteins (including SOX9 and SOX10) to mediate the nuclear export of SOX proteins across the nuclear envelope [28]. SOX9, as well as SOX10, harbors a perfect leucine-rich NES, which is a spaced and large hydrophobic NES consensus sequence Φ1-x_2–3_-Φ2-x_2–3_-Φ3-x-Φ4 (where Φ=L, I, V, F, M and x represents any amino acid). Previous studies have demonstrated that the functional leucine-rich NES on SOX9 and SOX10, and the CRM1-specific inhibitor LMB can block the nuclear export and then repress nucleocytoplasmic shuttling of these two SOXE proteins [26, 27, 32, 38, 39]. However, in all other SOX family transcription factors, including SRY, SOXB, SOXC, SOXD, SOXF proteins, whilst the Φ1, Φ2 and Φ4 residues are evolutionarily conserved, the Φ3 are absent [28]. A recent study have suggested that a CAG DNA microsatellite, encoding the variable glutamine-rich repeat domain, inserts into the rodent *Sry* gene. This results in a defective nuclear export signal in mouse *Sry* and enables rapid evolution [39]. Due to the lower affinities of CRM1 with nearly all other NES in non-subgroup E proteins, the nature of the nuclear export mechanisms in other SOX proteins has been a long-standing question for studies relating to SOX transcription factors [28, 32].

To solve this problem, we have used *Eriocheir sinensis* SOX14A and SOX14B proteins as the models. These are composed of two similarly imperfect leucine-rich NESs, as in other SOX proteins. In this study, we have demonstrated for the first time a functional NES located on SOX14A and SOX14B, which exists outside of the SOXE protein group (Figure 7A). We found that the nuclear export of SOX14A and SOX14B can be significantly inhibited by the CRM1-specific inhibitor LMB. In summary, the nuclear export of SOX14A/B can also be inhibited by LMB. These results indicate that SOX14A/B can be exported from the nucleus to the cytoplasm through a CRM1-dependent pathway (Figure 7B).

Meanwhile, we narrow down the nuclear export signal to the hydrophobic NES on the HMG box on SOX14A and SOX14B. The substitutions of leucines on NES also suggest the importance of the leucines or hydrophobic residues on NES during nuclear export. Our results clarify that the imperfect NES on other SOX proteins may indeed be functional and that the CRM1 mediated nuclear export pathway may be involved in the nucleocytoplasmic shuttling of non-subgroup E proteins. This result could shed a new light in the molecular mechanisms involved in the nuclear export of the SOX protein family.

## MATERIALS AND METHODS

### Cell culture and treatment

Human embryonic kidney (HEK) 293T cells were obtained from ATCC (ATCC No. CRL-3216), and were cultured in Dulbecco's modified Eagle's medium (GIBCO) supplemented with 10% heat-inactivated fetal bovine serum (GIBCO), with 2 mM _L-_glutamine (GIBICO), 1 mM sodium pyruvate (GIBCO), 0.1 mM minimal essential medium with nonessential amino acid (GIBCO), 100U/ml penicillin-streptomycin (GIBCO), and maintained at 37°C with 5% CO_2_. Where indicated, leptomycin B (Beyotime) was added 3h before cell fixation at a final concentration of 5.0 ng/ml. In this study, the methods were carried out in accordance with the approved guidelines and regulations. All experimental protocols were approved by the institutional ethic committee (College of Life Sciences, Zhejiang University, Hangzhou, China) according to the approved guidelines. The written informed consent was obtained from all subjects.

### Transient transfection

Transient transfections of plasmids into cultured 293T cells were performed using Lipofectamine 2000 reagent (Invitrogen), according to the manufacturer's instructions. The Lipofectamine 2000 complexes (for 24-well plate), including 500 ng plasmid DNA, 50μl Opti-MEM reduced serum medium (GIBCO) and 1μl Lipofectamine 2000 reagent (Invitrogen), were prepared and incubated in eppendorf tubes at room temperature for 5 min. The transfection components were added directly into the culture medium. The cultured cells were maintained for 24 hours and then harvested for further analyses.

### Plasmid construction

Full-length *Sox14a* and *Sox14b* were cloned from *Eriocheir sinensis* cDNA using Prime STAR HS DNA polymerase (Takara), and then cloned into pCMV-N-Flag vector (*Eco*R I and *Xho* I sites; Beyotime). Thereafter, EGFP and RFP were also cloned into pCMV-N-Flag-*Sox14a* and pCMV-N-Flag-*Sox14b* vectors, respectively (*Xho* I and *Xba* I sites; Beyotime).

N-terminal half (corresponding to amino acid 1-46, 1-34 of SOX14A and SOX14B, respectively), C-terminal half (corresponding to amino acid 125-423, 124-397), the HMG box (corresponding to amino acid 47-124, 35-123) and deletion of the HMG box (corresponding to amino acid 1-46 and 125-423, 1-34 and 124-397) of *E. sinensis* SOX14A *and* SOX14B sequences were designated as SOX14A and SOX14B deletion mutants (SOX14A/B ΔN, ΔC, HMG, ΔHMG), respectively. SOX14A/B deletion mutants (SOX14A/B ΔN, ΔC, HMG, ΔHMG) were individually generated by PCR-based cloning of the corresponding fragments. They were then tagged with EGFP/RFP epitope by cloning into pCMV-N-Flag vector. All the PCR primers used are listed in Supplementay Table 1.

### Oligo annealing and cloning

SOX14A/B HMG-related fragments HMG (HMG1/HMG2), ΔNLS1 (NES1/HMG2), ΔNLS2 (HMG1/NES1), ΔNLS1-2 (NES1/NES2), and double-strand oligos corresponding to SOX14A/B NES (amino acids 87-100, 108-122: EISKQLGKRWKN(M)LT) and to mutant SOX14A/B NES (2L-2A:EISKQAGKRWKN(M)AT and L-A: EISKQAGKRWKN(M)LT) were cloned into the backbone of the pCMV-N-Flag-EGFP/RFP vectors using the following protocols. These oligos were diluted in 100 μM and then phosphorylated and annealed by the T4 Polynucleotide Kinase (TaKaRa) following the parameters: 37°C for 30 min, 95°C for 5 min, and then ramp down to 15°C at 5°C/min. Finally, these annealed oligos were ligated into the pCMV-N-Flag-EGFP/RFP vector (*Eco*R I and *Xho* I sites; Beyotime), respectively. All the oligos used are listed in Supplementary Table 1.

### Sequence analysis, multiple sequence alignment and phylogenetic analysis

Sequence similarity analyses were performed using the Blast program at the National Center for Biotechnology Information (http://www.ncbi.nlm.nih.gov/blast). The open reading frame for *Sox14a* and *Sox14b* were determined using the ExPASy Translate Tool (http://web.expasy.org/translate/) and translated into putative amino acid sequences. Multiple sequence alignments of SOX14A and SOX14B genes and proteins were analyzed by the ClustalW algorithm and the phylogenetic trees were built using the Neighbor-Joining method of MEGA5.1 software with 1000 bootstrap replications.

### Western blot analysis

Cells were cultured in 12-well culture plate (Corning) and transfected with corresponding overexpression plasmids using Lipofectamine 2000 reagent (Invitrogen) and then cultured for another 24 hr. After transient transfection, about 10^6^ cells were collected and lysed in RIPA lysis buffer (Beyotime) at 4°C for 10 min. The cell lysates were mixed with 5×SDS loading buffer, and then heated and denatured at 100°C for 10 min. Total proteins were separated by 10% SDS-PAGE gel electrophoresis and transferred on a PVDF membrane. After incubated in 5% nonfat milk/TBST (150 mM NaCl, 50 mM Tris-HCl, 0.05% Tween-20, pH 7.4) for 1 hr at room temperature, membranes were incubated with anti-Flag antibody (Beyotime, 1:2000) for 1 hr at room temperature. After washed by TBST solution for 15 min, membranes were incubated with HRP-conjugated secondary antibodies (Beyotime, 1:2000) for 1 hr at room temperature. After washed by TBST solution for 15 min, the signals were visualized by chemiluminescence (Thermo Scientific). Anti-beta Actin antibody (1:2000, BBI) was used as a loading control.

### Fluorescence microscopy analysis

The 293T cells were cultured in cover-slips in the 24 well plates and fixed in 4% PFA/PBS for 10 min and stained with DAPI for 5 min. Slides were observed under a Zeiss confocal laser scanning microscope (CLSM710) using 63×1.4NA immersion oil lenses. The immunofluorescence images were acquired and exported using the ZEN2009 software (Zeiss). The gray scales and immunofluorescence intensities of the images, including immunoblot gels and fluorescence images, were quantified using the ImageJ software (NIH). All images were prepared using the Photoshop CS5.0 software (Adobe) according to the standard instructions.

### Statistical analysis

All experiments were repeated at least three independent times. For the analysis of fluorescence intensity, the Image J software and Microsoft Excel were used and the cells from three independent experiments were collected for analysis. For cell number quantifications, the ImageJ software and Adobe Photoshop CS5 software were used and more than 200 cells were counted on average, in each slide.

## SUPPLEMENTARY MATERIALS FIGURES AND TABLES



## Abbreviations

EGFP: enhanced green fluorescent protein
HMG: high mobility group
LMB: leptomycin B
NES: nuclear export signal
NLS: nuclear localization signals
RFP: red fluorescent protein
SOX: SRY-related HMG box
SRY: the sex determining region of chromosome Y.
