# Supplementary material for: Nucleocytoplasmic shuttling of SOX14A and SOX14B transcription factors
Source: Oncotarget. 2017 Feb 7;8(29):46955–68. doi: 10.18632/oncotarget.15134 (PMC5564536; doi:10.18632/oncotarget.15134)
Supplement: Supplementary file 1 [file oncotarget-08-46955-s001.pdf]

Nucleocytoplasmic shuttling of SOX14A and SOX14B transcription factors

SUPPLEMENTARY MATERIALS

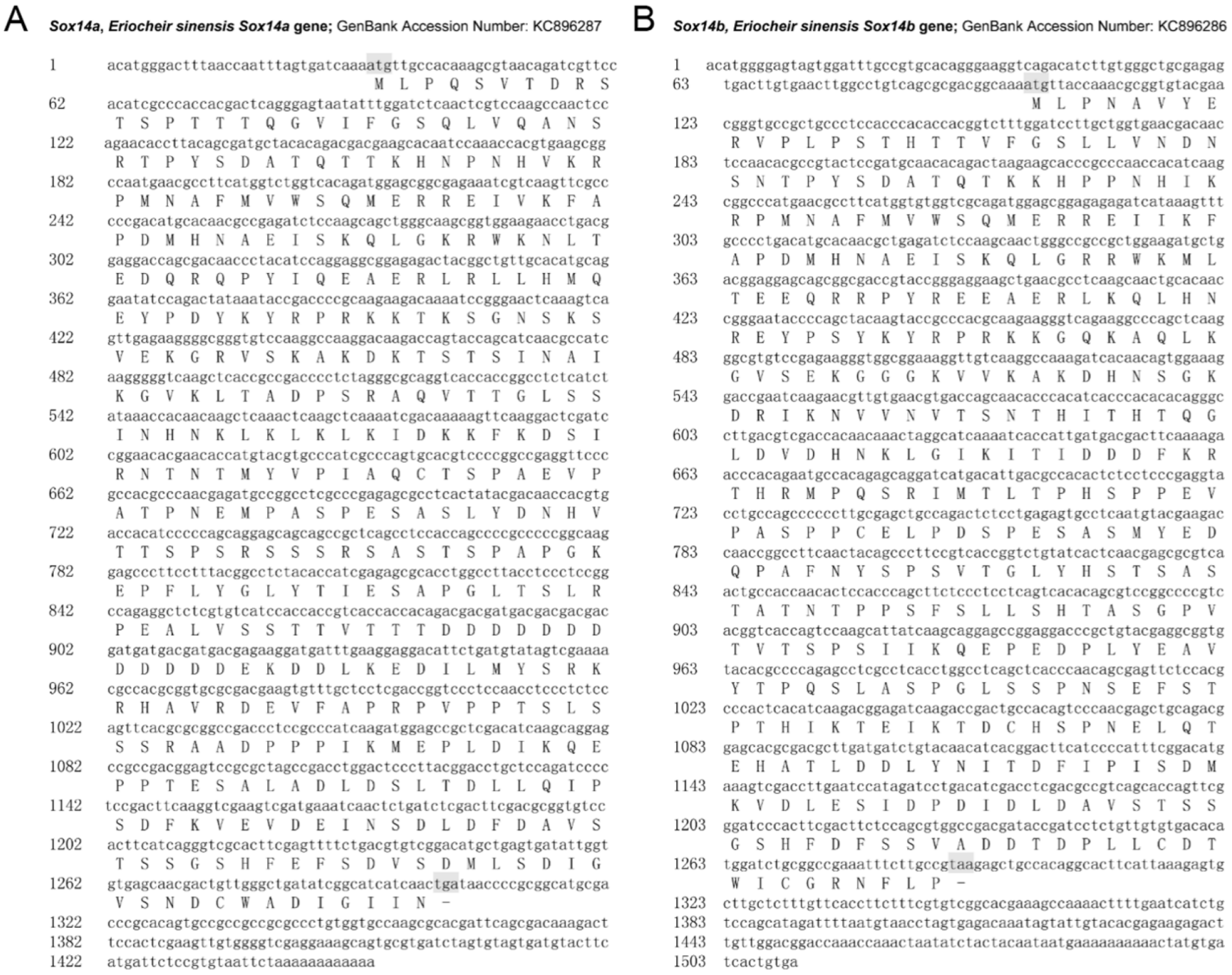

Supplementary Figure 1: The gene and protein sequences of *E. sinensis Sox14a* and *Sox14b*. A. The full-length sequences of *E. sinensis Sox14a* and *Sox14b* were cloned by degenerate PCR and RACE, respectively. The full-length *Sox14a* is 1473bp. *Sox14a* contains a 31 bp of the 5'untranslated region (UTR), a 1272 bp open reading frame (ORF) and a 170 bp of the 3' UTR (NCBI Accession Number KC896287). **B.** The full-length *Sox14b* is 1511bp. *Sox14b* contains 98 bp of the 5' UTR, a 1194 bp ORF and a 219 bp of the 3' UTR (NCBI Accession Number KC896286).

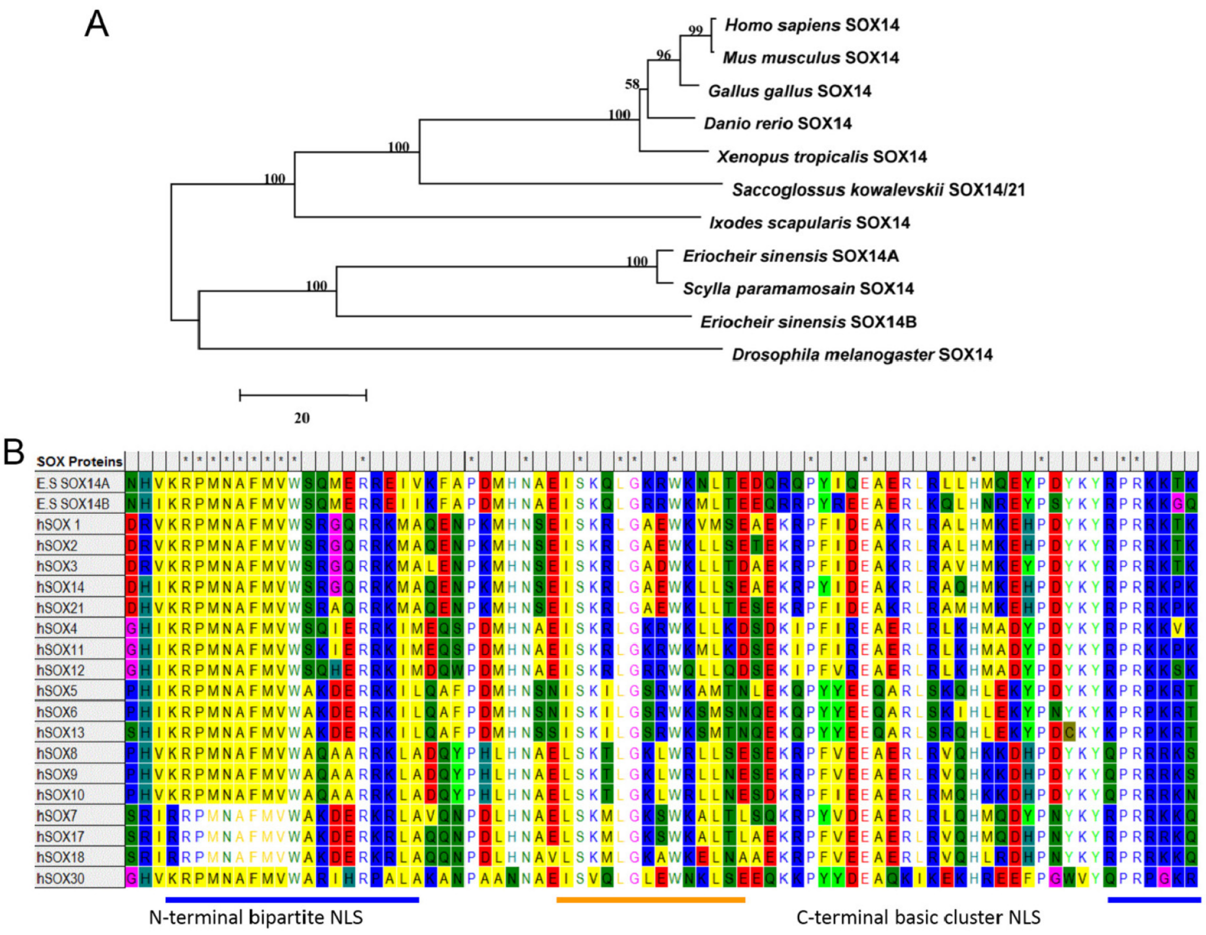

Supplementary Figure 2: Phylogenetic analyses of *E. sinensis* SOX14A/B proteins and multiple sequence alignment of the conserved HMG box. A. Neighbor-joining phelogenetic tree of *E. sinensis* SOX14A, SOX14B and other species SOX14 proteins. Numbers on the branches are the number of times, in a thousand bootstrap replications, that the two clades branched as sisters. The multiple sequence analysis indicates, with a high bootstrap support, that *E. sinensis* has two loci in the SOX14 clade. The NCBI Accession Numbers are listed as follows: *Homo sapiens* SOX14 (NP_004180), *Mus musculus* SOX14 (NP_035570), *Gallus gallus* SOX14 (BAA77265), *Danio rerio* SOX14 (NP_001032769), *Xenopus tropicalis* SOX14 (NP_001093703), *Saccoglossus kowalevskii* SOX14/21 (NP_001158461), *Ixodes scapularis* SOX14 (EEC02447), *Eriocheir sinensis* SOX14A (KC896287), *Eriocheir sinensis SOX14B* (KC896286), *Scylla paramamosain* SOX14 (ACM47362), and *Drosophila melanogaster* SOX14 (CAB64387). **B.** Multiple sequence alignments of the HMG box of the *E. sinensis* SOX14A/B and other SOX proteins. The NCBI Accession Numbers are listed as follows: *E. sinensis* SOX14A (KC896287), *E. sinensis SOX14B* (KC896286), *Homo sapiens* SOX1 (NP_005977), SOX2 (NP_003097), SOX3 (CAA50465), SOX14 (AAC95380), SOX21 (AAC95381), SOX4 (AAH72668), SOX11 (BAA88122), SOX12 (AAH67361), SOX5 (AAH60773), SOX6 (AAK26115), SOX13 (AAD50120), SOX8 (NP_055402), SOX9 (CAA86598), SOX10 (CAG30470), SOX7 (CAC84226), SOX17 (BAB83867), SOX18 (BAA94874), SOX30 (BAA37146).

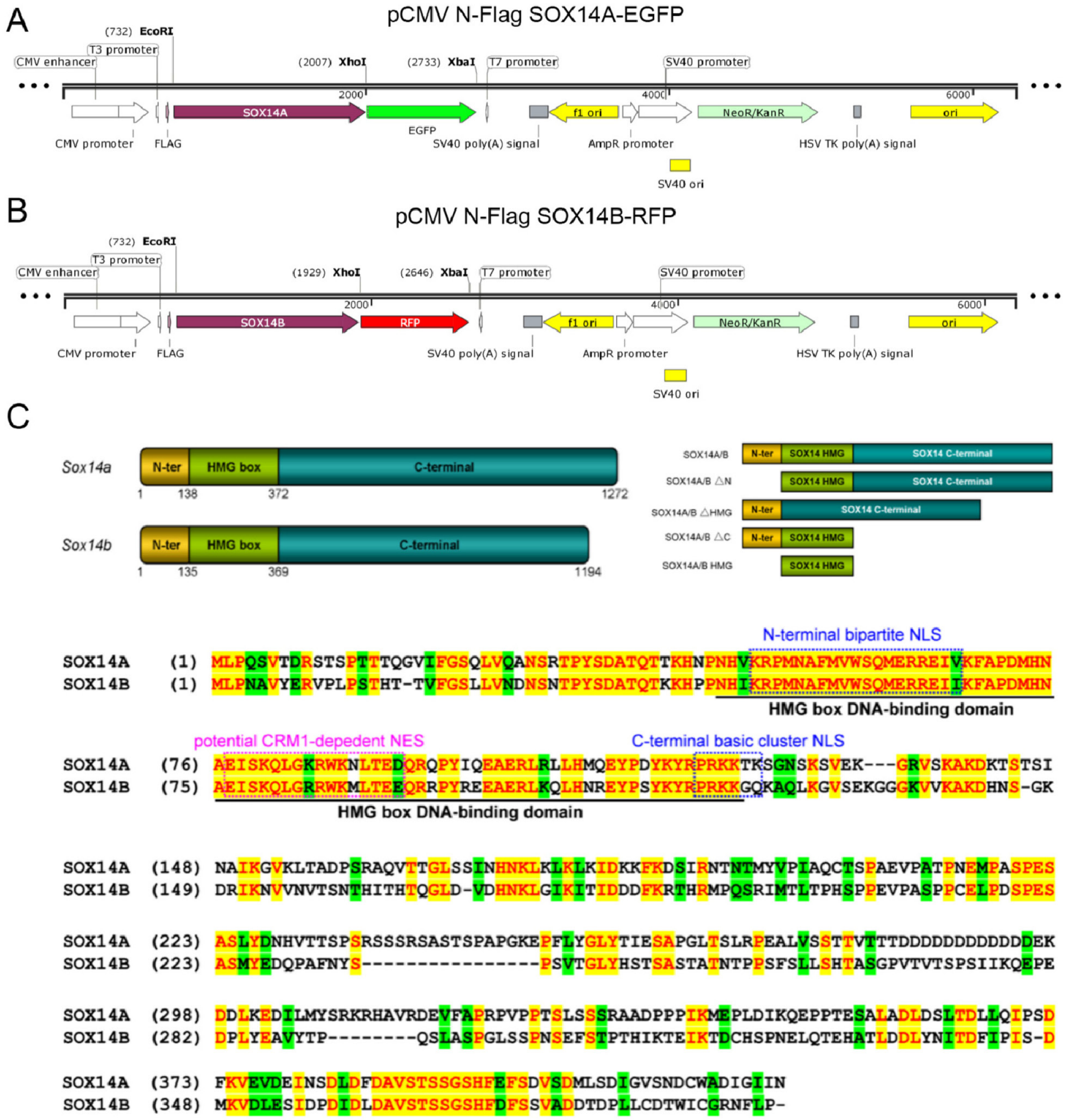

Supplementary Figure 3: Schematic representations of the designed clones of *E. sinensis Sox14a* and *Sox14b*, and the sequence alignment of these two transcription factors. **A** and **B.** The *E. sinensis Sox14a* and *Sox14b* were cloned into the backbone of pCMV-N-Flag vector. The other features of the expression vectors were also shown in the figures. **C.** Schematic representations of SOX14A/B and mutant fusion proteins. The sequence alignment of *E. sinensis* SOX14A and SOX14B proteins. The N-terminal bipartite NLS, C-terminal basic cluster NLS and potential CRM1-dependent NES were indicated.

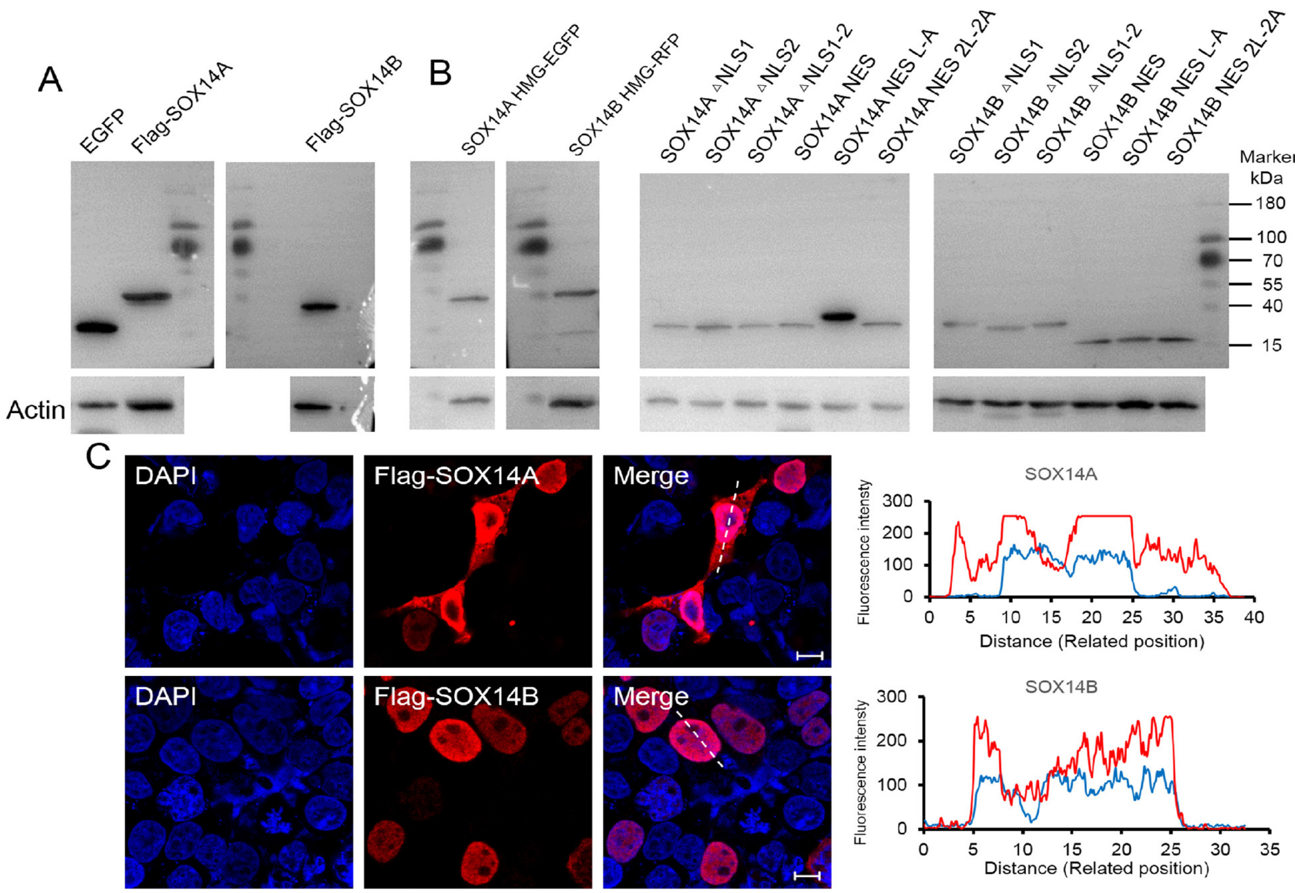

Supplementary Figure 4: Western blot analysis of the SOX14A/B fusion proteins and subcellular distributions of Flag-SOX14A/B proteins. **A** and **B.** Western blot analysis of the SOX14A and SOX14B mutant fusion proteins after transfected into 293T cells for 24 h. The fusion proteins were detected using anti-Flag antibody. Beta-Actin was used as loading control. **C.** The subcellular localizations of Flag-SOX14A and Flag-SOX14B in 293T cells. The 293T cells were transiently transfected with Flag-SOX14A and Flag-SOX14B, and then maintained at 37°C for 24 h. The Flag-tagged SOX14A/B fusion proteins (red) were labeled by the mouse anti-Flag antibodies by the immunofluorescence assays. The nuclei were stained by DAPI (blue). The fluorescence intensities of Flag-SOX14A/B protein transfected cells (white dot line) were analyzed using the ImageJ software. The X axis means the relative position. The Y axis means the relative fluorescence intensities.

Supplementary Table 1: Primers used in this study

Primers name	Sequences (5'-3')	Notes
Primers used in the construction of EGFP/RFP fusion proteins		
EGFP F1(*Xho*I)	CCG**CTCGAG**ATGGTGAGCAAGGGCGAGGAGCT	
EGFP R1(*Xba*I)	TGC**TCTAGA**TTACTTGTACAGCTCGTCCATGCCG	
RFP F1(*Xho*I)	CCG**CTCGAG**ATGGTGAGCAAGGGCGAGGAGGA	
RFP R1(*Xba*I)	TGC**TCTAGA**CTACTTGTACAGCTCGTCCATGCCGC	
Primers used in the construction of SOX14A or mutant-EGFP fusion proteins		
SOX14A F1(*Eco*RI)	CCG**GAATTC**ATGTTGCCACAAAGCGTAACAGATC	
SOX14A R1(*Xho*I)	CCG**CTCGAG**GTTGATGATGCCGATATCAGCCCA	
SOX14A F2(*Eco*RI)	CCG**GAATTC**CACGTGAAGCGGCCAATGAAC	
SOX14A R2(*Xho*I)	CCG**CTCGAG**CCCGGATTTTGTCTTCTTGCGG	
SOX14A F3(*Xho*I)	CCG**CTCGAG**AACTCAAAGTCAGTTGAGAAGGGGC	
SOX14A R3(*Xho*I)	CCG**CTCGAG**GTTTGGATTGTGCTTCGTCGTCTG	
Primers used in the construction of SOX14B or mutant-RFP fusion proteins		
SOX14B F1(*Eco*RI)	CCG**GAATTC**ATGTTACCAAACGCGGTGTACGAAC	
SOX14B R1(*Xho*I)	CCG**CTCGAG**CGGCAAGAAATTTCGGCCGCAGAT	
SOX14B F2(*Eco*RI)	CCG**GAATTC**AACCACATCAAGCGGCCCATG	
SOX14B R2(*Xho*I)	CCG**CTCGAG**CTTCTGACCCTTCTTGCGTGGG	
SOX14B F3(*Xho*I)	CCG**CTCGAG**GCCCAGCTCAAGGGCGTGTC	
SOX14B R3(*Xho*I)	CCG**CTCGAG**GGGCGGGTGCTTCTTAGTCTGTG	
Primers used in the study of the nuclear import of SOX14A/B HMG box or ΔNLS mutant fusion proteins		
SOX14A NES F1 (*Eco*RI)	CCG**GAATTC**AAGTTCGCCCCCGACATGCAC	
SOX14A NES R1 (*Xho*I)	CCG**CTCGAG**GTATTTATAGTCTGGATATTCCTGCATG	
SOX14B NES F1 (*Eco*RI)	CCG**GAATTC**ATAAAGTTTGCCCCTGACATGCAC	
SOX14B NES R1 (*Xho*I)	CCG**CTCGAG**GTACTTGTAGCTGGGGTATTCCCG	
Primers used in the study of the nuclear export of SOX14A NES or NES mutant fusion proteins		
SOX14A NES F2 (*Eco*RI)	**AATTC**GAGATCTCCAAGCAGCTGGGCAAGCGGTGGAAGAACCTGACG**C**	
SOX14A NES R2 (*Xho*I)	**TCGAG**CGTCAGGTTCTTCCACCGCTTGCCCAGCTGCTTGGAGATCTC**G**	
SOX14A NES F3 (*Eco*RI)	**AATTC**GAGATCTCCAAGCAGGCCGGCAAGCGGTGGAAGAACGCCACG**C**	2L-2A
SOX14A NES R3 (*Xho*I)	**TCGAG**CGTGGCGTTCTTCCACCGCTTGCCGGCCTGCTTGGAGATCTC**G**	2L-2A
SOX14A NES F4 (*Eco*RI)	**AATTC**GAGATCTCCAAGCAGGCCGGCAAGCGGTGGAAGAACCTGACG**C**	L-A
SOX14A NES R4 (*Xho*I)	**TCGAG**CGTCAGGTTCTTCCACCGCTTGCCGGCCTGCTTGGAGATCTC**G**	L-A
Primers used in the study of nuclear export of SOX14B NES or NES mutant fusion proteins		
SOX14B NES F2 (*Eco*RI)	**AATTC**GAGATCTCCAAGCAACTGGGCCGCCGCTGGAAGATGCTGACG**C**	
SOX14B NES R2 (*Xho*I)	**TCGAG**CGTCAGCATCTTCCAGCGGCGGCCCAGTTGCTTGGAGATCTC**G**	
SOX14B NES F3 (*Eco*RI)	**AATTC**GAGATCTCCAAGCAAGCCGGCCGCCGCTGGAAGATGGCCACG**C**	2L-2A
SOX14B NES R3 (*Xho*I)	**TCGAG**CGTGGCCATCTTCCAGCGGCGGCCGGCTTGCTTGGAGATCTC**G**	2L-2A
SOX14B NES F4 (*Eco*RI)	**AATTC**GAGATCTCCAAGCAAGCCGGCCGCCGCTGGAAGATGCTGACG**C**	L-A
SOX14B NES R4 (*Xho*I)	**TCGAG**CGTCAGCATCTTCCAGCGGCGGCCGGCTTGCTTGGAGATCTC**G**	L-A

The words indicated in bold are the cut sites of restriction enzymes.
